# RatDEGdb: a knowledge base of differentially expressed genes in the rat as a model object in biomedical research

**DOI:** 10.18699/VJGB-23-92

**Published:** 2023-12

**Authors:** I.V. Chadaeva, S.V. Filonov, K.A. Zolotareva, B.M. Khandaev, N.I. Ershov, N.L. Podkolodnyy, R.V. Kozhemyakina, D.A. Rasskazov, A. G. Bogomolov, E.Yu. Kondratyuk, N.V. Klimova, S.G. Shikhevich, M.A. Ryazanova, L.A. Fedoseeva, О.Е. Redina, O.S. Kozhevnikova, N.A. Stefanova, N.G. Kolosova, A.L. Markel, M.P. Ponomarenko, D.Yu. Oshchepkov

**Affiliations:** Institute of Cytology and Genetics of the Siberian Branch of the Russian Academy of Sciences, Novosibirsk, Russia; Institute of Cytology and Genetics of the Siberian Branch of the Russian Academy of Sciences, Novosibirsk, Russia Novosibirsk State University, Novosibirsk, Russia; Institute of Cytology and Genetics of the Siberian Branch of the Russian Academy of Sciences, Novosibirsk, Russia; Institute of Cytology and Genetics of the Siberian Branch of the Russian Academy of Sciences, Novosibirsk, Russia; Institute of Cytology and Genetics of the Siberian Branch of the Russian Academy of Sciences, Novosibirsk, Russia; Institute of Cytology and Genetics of the Siberian Branch of the Russian Academy of Sciences, Novosibirsk, Russia Novosibirsk State University, Novosibirsk, Russia; Institute of Cytology and Genetics of the Siberian Branch of the Russian Academy of Sciences, Novosibirsk, Russia; Institute of Cytology and Genetics of the Siberian Branch of the Russian Academy of Sciences, Novosibirsk, Russia; Institute of Cytology and Genetics of the Siberian Branch of the Russian Academy of Sciences, Novosibirsk, Russia; Institute of Cytology and Genetics of the Siberian Branch of the Russian Academy of Sciences, Novosibirsk, Russia Siberian Federal Scientific Centre of Agro-BioTechnologies of the Russian Academy of Sciences, Krasnoobsk, Novosibirsk region, Russia; Institute of Cytology and Genetics of the Siberian Branch of the Russian Academy of Sciences, Novosibirsk, Russia; Institute of Cytology and Genetics of the Siberian Branch of the Russian Academy of Sciences, Novosibirsk, Russia; Institute of Cytology and Genetics of the Siberian Branch of the Russian Academy of Sciences, Novosibirsk, Russia; Institute of Cytology and Genetics of the Siberian Branch of the Russian Academy of Sciences, Novosibirsk, Russia; Institute of Cytology and Genetics of the Siberian Branch of the Russian Academy of Sciences, Novosibirsk, Russia; Institute of Cytology and Genetics of the Siberian Branch of the Russian Academy of Sciences, Novosibirsk, Russia; Institute of Cytology and Genetics of the Siberian Branch of the Russian Academy of Sciences, Novosibirsk, Russia; Institute of Cytology and Genetics of the Siberian Branch of the Russian Academy of Sciences, Novosibirsk, Russia; Institute of Cytology and Genetics of the Siberian Branch of the Russian Academy of Sciences, Novosibirsk, Russia Novosibirsk State University, Novosibirsk, Russia; Institute of Cytology and Genetics of the Siberian Branch of the Russian Academy of Sciences, Novosibirsk, Russia; Institute of Cytology and Genetics of the Siberian Branch of the Russian Academy of Sciences, Novosibirsk, Russia

**Keywords:** knowledge base, DEG, Rattus norvegicus, animal models of human diseases, neurodegeneration, Alzheimer’s disease, hypertension, premature aging, psychopathological states, catatonic syndrome, epilepsy, aggression, RNA-seq, PCR, microarrays, база знаний, ДЭГ, крысы Rattus norvegicus, животные модели болезней человека, гипертоническая болезнь, преждевременное старение, психопатологические состояния, нейродегенерация, болезнь Альцгеймера, кататонический синдром, эпилепсия, агрессивность, RNA-seq, ПЦР, микрочипы

## Abstract

The animal models used in biomedical research cover virtually every human disease. RatDEGdb, a knowledge base of the differentially expressed genes (DEGs) of the rat as a model object in biomedical research is a collection of published data on gene expression in rat strains simulating arterial hypertension, age-related diseases, psychopathological conditions and other human afflictions. The current release contains information on 25,101 DEGs representing 14,320 unique rat genes that change transcription levels in 21 tissues of 10 genetic rat strains used as models of 11 human diseases based on 45 original scientific papers. RatDEGdb is novel in that, unlike any other biomedical database, it offers the manually curated annotations of DEGs in model rats with the use of independent clinical data on equal changes in the expression of homologous genes revealed in people with pathologies. The rat DEGs put in RatDEGdb were annotated with equal changes in the expression of their human homologs in affected people. In its current release, RatDEGdb contains 94,873 such annotations for 321 human genes in 836 diseases based on 959 original scientific papers found in the current PubMed. RatDEGdb may be interesting first of all to human geneticists, molecular biologists, clinical physicians, genetic advisors as well as experts in biopharmaceutics, bioinformatics and personalized genomics. RatDEGdb is publicly available at https://www.sysbio.ru/RatDEGdb.

## Abstract (ru)

Животные модели, используемые в биомедицинских исследованиях, в настоящее время охватывают практически весь известный спектр заболеваний человека. База знаний RatDEGdb по дифференциально экспрессирующимся генам (ДЭГ) крысы как модельного объекта в биомедицинских исследованиях представляет собой коллекцию опубликованных данных по экспрессии генов у крыс разных линий, предназначенных для изучения артериальной гипертонии, болезней пожилого возраста, психопатологических состояний и других заболеваний человека. Текущий выпуск RatDEGdb содержит 25 101 ДЭГ, представляющих 14 320 уникальных генов крысы, которые изменяют уровень транскрипции в 21 ткани 10 генетических линий крысы в качестве моделей 11 заболеваний человека согласно 45 оригинальным научным статьям. Новшество RatDEGdb по сравнению с другими биомедицинскими базами данных заключается в курируемой аннотации отклонений ДЭГ крысы как модельного объекта с использованием независимых клинических данных об однонаправленных изменениях экспрессии гомологичных генов, выявленных у людей при различных патологиях. Собранные ДЭГ крыс были аннотированы однонаправленными изменениями экспрессии гомологичных им генов человека у больных людей относительно здоровых. К настоящему времени выпуск RatDEGdb содержит 94 873 такие аннотации для 321 гена человека при 836 заболеваниях согласно 959 оригинальным научным статьям, найденным в текущем выпуске базы данных PubMed. Представленная база знаний может быть интересна в первую очередь специалистам по генетике человека, молекулярным биологам, клиницистам и генетическим консультантам, а также специалистам в области биофармацевтики, биоинформатики и персонализированной геномики. RatDEGdb является общедоступной (https://www.sysbio.ru/RatDEGdb).

## Introduction

The animal models required for understanding the physiological, genetic and epigenetic mechanisms regulating evolutionarily fixed phenotypic traits of an organism are supposed to perfectly mimic the symptoms of the pathology being studied and to conform to strict criteria (Gryksa et al., 2023). The most popular animal models are rats and mice, with dozens of thousands of laboratory strains in use (Gayday E.A., Gayday D.S., 2019).

The first inbred rat strain was developed in 1906 in the Wistar Institute (Philadelphia, USA), about the time that mice came to the laboratory settings. Nevertheless, the mouse has become the model of choice for research into mammalian genetics, and the rat, into physiology and biomedicine. Laboratory rats have certain advantages over mice: rats are larger and therefore submit more tissue for analyses. Large organs make surgical procedures more manageable and rather small anatomical structures easier to dissect.

A low maintenance and cheap species, the rat (Rattus norvegicus) has become a convenient object in numerous biomedical research studies (Carter et al., 2020; Modlinska, Pisula, 2020). Rats are recommended for use as model animals in studying aging, hypertension, catalepsy etc. (Carter et al., 2020; Martín-Carro et al., 2023).

There are generally acknowledged differences between wild and laboratory rats. For example, laboratory rats are noted for smaller adrenals and preputial glands, earlier puberty, lack of seasonality of reproduction and higher fertility than have their wild conspecifics. In addition, the rat and human genomes share a 90 % identity (Gibbs et al., 2004). Thus, the genetic strains of laboratory rats simulating human pathologies have been developed: for example, the Zucker strain for human obesity, hypertension, type II diabetes and heart disease (Schmidt, 2002); the reelin-deficient shaking rat Kawasaki for schizophrenia and autism (Aikawa et al., 1988); and the Brattleboro strain for hypothalamic diabetes insipidus (Ideno et al., 2003). To date, there are about 1,000 inbred strains of laboratory rats developed by genetic breeding that have “fixed” alleles for natural diseases (Greenhouse et al., 1990), such as mental disorders (Taylor et al., 2002), depression (Bay et al., 2020) and chronic renal failure (Zhang H.F. et al., 2019). The Wistar and Sprague-Dawley strains are the most commonly used laboratory rats (Sengupta, 2013). At present, the search of PubMed (Lu, 2011) with “rats biomedical model” as a search string returns the annotations of 19,555 original scientific papers, which lends support to the relevance of the subject.

To contribute to the effort, several rat strains simulating human diseases have been developed in the Institute of Cytology and Genetics of the Siberian Branch of the Russian Academy of Sciences. Thus, the ISIAH rats are characterized by an increased arterial blood pressure and used for studying the causes and treatments of hypertension in humans (Markel, 1992; Markel et al., 1999; Fedoseeva et al., 2016a, 2019; Klimov et al., 2016; Ryazanova et al., 2016), the OXYS rats represent a unique selection-based model of premature ageing and associated diseases (Kozhevnikova et al., 2013; Kolosova et al., 2014; Perepechaeva et al., 2014; Stefanova et al., 2018, 2019; Stefanova, Kolosova, 2023), rats with pendulum-like movements (the PM strain) with stereotypies and audiogenic epilepsy, and rats with genetic catatonia (the GC rats), a syndrome observed in patients with mental disorders, including schizophrenia (Barykina et al., 1983; Kolpakov et al., 2004; Ryazanova et al., 2017, 2023).

Changes in the expression of the genes associated with a disease of interest have been studied in the model rats by semi-quantitative real-time PCR of separate key genes or by profiling transcriptomes by next-generation sequencing or by use of microarrays. This effort has created a large body of data on the differentially expressed genes (DEGs) significantly associated with diseases, and it has become possible to collect, perform comparative analyses on and systematize the results obtained from these or similar experiments with the use of bioinformatics technologies. This has enabled the development of specialized databases and knowledge bases.

The aim of this work was to create a knowledge base containing information on DEGs of various rat strains developed, first of all, in the Institute of Cytology and Genetics of the Siberian Branch of the Russian Academy of Sciences as well as those developed in a range of Russia’s and other scientific organizations. This knowledge base is freely available at https://www.sysbio.ru/RatDEGdb.

## Materials and methods

Experimental animals. We performed in vivo experiments on 12 adult male gray rat (Rattus norvegicus) from two outbred strains resulting from genetic breeding for more than 90 generations in two directions (Belyaev, Borodin, 1982): one for increased aggressive behavior towards humans (the aggressive strain) and one for decreased (the tame strain). The animals were kept in standard conditions at the Conventional Animal Facility of the Institute of Cytology and Genetics of the Siberian Branch of the Russian Academy of Sciences (Novosibirsk, Russia) as groups by four in 50×33×20 cm cages at an adjustable light/dark cycle (12 light:12 dark) and had free access to water and complete feed.

The test subjects were two-month-old individuals, each weighing 250–270 g, from unrelated litters. Within the first 4 hours of the light phase of the diurnal light-dark cycle, each animal’s level of tameness/aggression was measured in the “glove” test as the reaction to a gloved hand and was scored from “–4” (most aggressive) to “+4” (most friendly), according to Plyusnina and Oskina (1997). Upon the completion of this test, the animals were put back to their home cages and kept in standard conditions for one week, to reduce possible effects that the “glove” test might have on gene expression, at which point the animals were euthanized and hypothalamus specimens were prepared according to the brain atlas of Paxinos and Watson (2013). Samples were placed in liquid nitrogen for transportation and further storage at –70 °C until use. The protocol of experiments was approved by the Commission on Bioethics at the Institute of Cytology and Genetics of the Siberian Branch of the Russian Academy of Sciences (resolution No. 97 as of October 28, 2021).

Measurement of the hypothalamic mRNA levels of the Asmtl gene in tame and aggressive male gray rats by semi-quantitative PCR. To measure mRNA levels by semiquantitative real-time polymerase chain reaction, hypothalamic RNA was isolated from six aggressive rats (n = 6) and six tame rats (n = 6), each specimen weighing ~100 mg. Total RNA was isolated using TRIzol™ (Invitrogen, #15596018) and purified using magnetic beads in the Agencourt RNAClean XP Kit (Beckman, #A63987). Purified RNA was quantified using a Qubit™ 2.0 fluorimeter (Invitrogen/Life Technologies) and a Qubit™ RNA High-Sensitivity Assay Kit (Invitrogen #In=Q32852). Next, we synthesized cDNA using the Reverse Transcription Kit (Syntol, #OT-1).

The oligonucleotide primers for each gene in question were designed using the web service PrimerBLAST (Ye et al., 2012) (Table 1). Real-time PCR was carried out using the EVA Green I kit in three technical replicates in a LightCycler® 96 operated in the automatic mode, according to the manufacturer’s instruction (Roche, Switzerland). The efficiency of the polymerase chain reaction was determined by serial cDNA dilutions (standards).

**Table 1.**
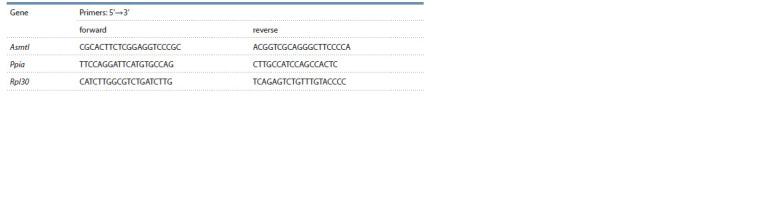
Primers for quantitative real-time polymerase chain reaction (qPCR) Notе. Primers were selected using the freely available web service PrimerBLAST (Ye et al., 2012). Rat genes: Asmtl, acetylserotonin O-methyltransferase like; Ppia, peptidylprolyl isomerase A; Rpl30, ribosomal protein L30. qPCR, quantitative real-time polymerase chain reaction using two reference genes as recommended by Bustin et al. (2009). The reference genes of our choice were Ppia (Gholami et al., 2017) and Rpl30 (Penning et al., 2007) (for experimental substantiation, see our previous works (Chadaeva et al., 2021)).

The human gene ASMT encodes acetylserotonin O-methyltransferase, a key enzyme in the synthesis of melatonin, one of the hormones that regulate the molecular and genetic processes in the entire organism, including circadian rhythms as well as cancer protective (Lv et al., 2019), anti-inflammatory, and immunomediatory mechanisms (Li G. et al., 2021). That is why the mRNA level of its rat homolog, Asmtl, in the hypothalamus of adult tame and aggressive male rats used as model animals in the biomedical studies of increased aggression was heuristically chosen as the quantity to be found by semiquantitative real-time PCR (real-time PCR) in its first run. As was recommended by Bustin and the co-workers (2009), the Asmtl mRNA values were normalized to the mRNA levels of two comparison genes, Ppia (Gholami et al., 2017) and Rpl30 (Penning et al., 2007). The relevance of Ppia and Rpl30 as the comparison genes in the experimental identification of DEGs in the hypothalamus of these aggressive and tame rat strains by real-time PCR was demonstrated in one of our previous works (Chadaeva et al., 2021).

RatDEGdb: the knowledge base. The observed lower hypothalamic levels of the Asmtl gene in the adult aggressive and tame male rats were checked against clinical data suggesting that lower levels of the protein encoded by its human homologs ASMT and ASMTL were in patients with various diseases than in otherwise healthy individuals. The results of this comparison were presented in an Excel-compatible, flat text format and then converted to RatDEGdb containing information about differential gene expression in the rat used as a model animal in biomedical research (URL=https:// www.sysbio.ru/RatDEGdb). The conversion was performed using MariaDB 10.2.12, a freely available database (MariaDB Corp AB, Finland).

Likewise, Lu (2011) submitted a representative selection of PubMed publications telling about the current diversity of laboratory rat strains used as biomedical models simulating human diseases and about experimental methods to assess differential gene expression with. Next, all rat DEGs in this selection of papers were documented and uploaded to RatDEGdb together with their supervised annotations, using an algorithm similar to the one described above for hypothalamic deficiency of Asmtl in aggressive rats. The lists of homologous genes were taken from the paralogs section of the GeneCards database (Stelzer et al., 2016). RatDEGdb includes the statistical significance of each DEG according to the estimates provided in the papers as referenced.

Statistical analysis of the differential expression of the Asmtl gene in the hypothalamus of the tame and aggressive rats used as an animal model of human aggressive behavior was performed using the menu “Statistics → Nonparamet- ric → Mann–Whitney test” in STATISTICA (StatSoft™, USA), when two independent statistical criteria are being assessed at once: the nonparametric Mann–Whitney U test and the parametric test Fisher’s Z, to assess the sustainability of results.

## Results

Lower hypothalamic Asmtl mRNA levels in aggressive than in tame rats

**Fig. 1.**
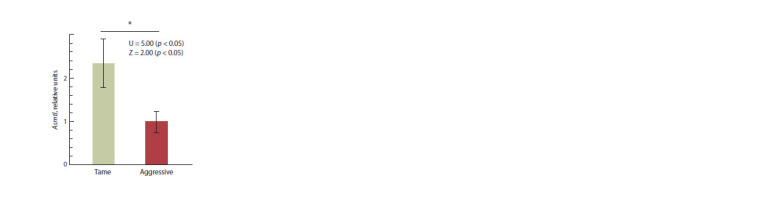
Statistically significant differences in hypothalamic Asmtl expression levels between tame and aggressive adult male rats. * Significance level p < 0.05 according to two independent statistical criteria: the nonparametric Mann–Whitney U test and the parametric test Fisher’s Z, which reflects the sustainability of assessment results for Asmtl as a differentially expressed gene (DEG) in aggressive versus tame rats.

Asmtl mRNA levels in the hypothalamus as measured and compared between the aggressive and tame rats are presented in Table 2. As can be seen from Figure 1, significantly lower Asmtl mRNA levels were in the aggressive than in tame rats in the settings of this experiment ( p < 0.05; the Mann–Whitney U test and Fisher’s Z).

**Table 2.**
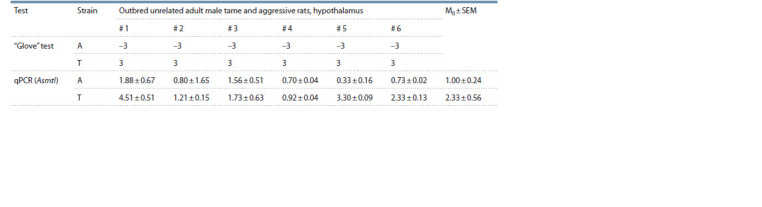
Experimental data on “glove” test behavior and Asmtl mRNA levels for 12 adult male rats Notе. see Notes to Table 1. Rat strain: A, aggressive rats (n = 6); T, tame rats (n = 6). Tests: “glove” test, in which each rat was scored from “–4” (most aggressive) to “+4” (most friendly), according to a work by Plyusnina and Oskina (1997); Asmtl expression levels, M0 ± SEM, estimates of the mean ± standard error of the mean from three technical replicates, with a LightCycler® 96 operated in the automatic mode (Roche, Switzerland).

Clinical manifestations of human ASMTL and ASMT deficiency

Table 3 presents the PubMed search results, with search terms (Lu, 2011) relating to human diseases associated with low expression levels of the ASMTL gene and its human paralog, ASMT. Line 1: the Asmt-deleted mouse models of human diseases (Trent et al., 2013) suggest a neurodevelopmental problem in the form of attention-deficit/hyperactivity disorder in combination with externalization symptoms (aggressive behavior) in children (Kang et al., 2023).

**Table 3.**
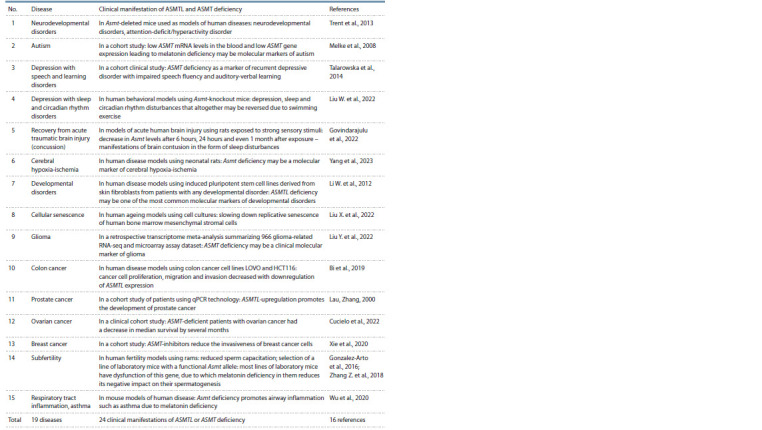
Clinical manifestation of deficiencies in ASMTL and in its human paralogue ASMT in human diseases according to the current release of the RatDEGdb knowledge base

Line 2: ASMT deficiency is a molecular marker of autism, according to Melke and co-workers (2008), while a recent survey of teenagers above 12 years of age with autism spectrum disorders and epilepsy in past medical history revealed their inclination to aggression (Gaitanis et al., 2023).

These two examples are in favor of rather than against the low expression levels of the human genes ASMTL and ASMT representing, at least, combined molecular characteristics of the predisposition to some forms of aggressive behavior.

Finally, as can be seen from Table 3, these human genes were expressed at low levels among candidate molecular markers of a wide range of human diseases not associated with aggression: depression (Talarowska et al., 2014), developmental abnormalities (LiW. et al., 2012), brain injury (Govindarajulu et al., 2022; Yang et al., 2023), cell aging (Liu X. et al., 2022), cancer (Bi et al., 2019; Lau, Zhang, 2000; Xie et al., 2020; Cucielo et al., 2022; Liu Y. et al., 2022), infertility (Gonzalez-Arto et al., 2016; Zhang Z. et al., 2018) and asthma (Wu et al., 2020).

Put together, these findings reflect the fact that ASMT gene encoding the melatonin synthesis enzyme acetylserotonin O-methyltransferase is one of the key hormones involved in the regulation of molecular and genetic processes in all human body in general including aggression (Melke et al., 2008; Trent et al., 2013; Gaitanis et al., 2023; Kang et al., 2023), depression (Talarowska et al., 2014), ontogenesis (LiW. et al., 2012; Zhang Z. et al., 2018), wound healing (Govindarajulu et al., 2022; Yang et al., 2023), ageing (Liu X. et al., 2022) oncoprotector (Lv et al., 2019), anti-inflammatory and immunomediatory mechanisms (Li G. et al., 2021).

RatDEGdb: the knowledge base

**Fig. 2.**
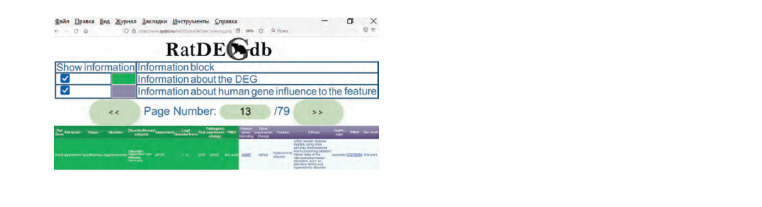
A sample entry in RatDEGdb documents original experimental data on Asmtl deficiency in the hypothalamus of aggressive rats compared to the tame rats as a biomedical model of aggressive behavior in human diseases (see Fig. 1 and Table 2) together with their annotation (see Table 3: first row) using independent data on low expression levels of its human homolog ASMT in patients with hyperactivity disorders according to an Asmt-deleted mouse model of human disease (Trent et al., 2013).

Figure 2 shows how RatDEGdb compares the hypothalamic level of Asmtl in the aggressive rat strain with that in the tame. Here aggression is considered to be a comorbid symptom in human diseases such as thalassemia, obesity and carcinoma (for review, see Chadaeva et al., 2016). Consequently, RatDEGdb (see Fig. 1 and Table 2) integrated data on low hypothalamic levels of the Asmtl gene in the aggressive rats and low levels of its human homolog ASMTL as found in patients with neurodevelopmental problems in the form of attention-deficit/hyperactivity disorder using an Asmt-deleted mouse model (Trent et al., 2013) (see Table 3).

The current release contains information on DEGs in ten genetic rat strains used as models of 11 human pathologies (Tables 4–6). As can be seen in the bottom lines of these tables, RatDEGdb now contains information on 25,101 DEGs representing 14,320 unique rat genes that change transcription levels in 21 tissues of 10 genetic rat strains used as models of 11 human diseases based on 45 original scientific papers referenced in the rightmost column of Tables 4–6. These rat DEGs were annotated with information about equal changes in the expression levels of their human homologs in affected people. In total, the current release contains 94,873 such annotations for 321 human genes in 836 diseases based on 959 PubMed publications (Lu, 2011). Thus, RatDEGdb is unique in that the manual curation of the annotation of DEGs of the rat as a model object simulating human pathology uses independent clinical data, which none of other biomedical databases does.

## Discussion

The elementary step in filling RatDEGdb with data can be seen in Tables 1–3 and Figures 1–2, with the Asmtl (acetylserotonin O-methyltransferase like) gene as an example. The hypothalamic expression of this gene was profiled and compared between aggressive and tame rats used as model animals in human aggression research. Results of the analysis of this gene by real-time PCR are provided. These results were annotated using PubMed papers (Lu, 2011) about equal changes in the expression levels of its human homologs ASMTL and ASMT in patients. Then this annotation of the Asmtl gene differentially expressed in the hypothalamus of the aggressive and tame rats was supplemented with PCR-, RNA-seq- and microarray-based information on all DEGs in the rat used as a model object in biomedical research. Next, the uncharacterized, unannotated, predicted, and not protein-encoding genes were dropped. Finally, we annotated the remaining rat DEGs with publicly available works about the clinical manifestations of equal changes in the expression levels of their human homologs in patients, put these annotations together as the RatDEGdb the knowledge base, and made it freely available at https://www.sysbio.ru/RatDEGdb.

Figures 1 and 2 show how RatDEGdb characterizes the DEGs of various breeding-based rat strains primarily developed in the Institute of Cytology and Genetics of the Siberian Branch of the Russian Academy of Sciences (Novosibirsk, Russia). The ISIAH rats were used as model animals in the biomedical studies of stress-induced arterial hypertension, as summarized in Tables 4 and 5. The same tables show that that OXYS rats were used for studying age-related diseases and ageing processes; and GC rats, for studying psychopathological conditions (see Table 4). In addition, tame and aggressive rat strains were used for studying animal domestication (Plyusnina, Oskina, 1997; Gulevich et al., 2019; Chadaeva et al., 2021) and aggression (Popova et al., 2010) as symptoms of obesity and thalassemia (Chadaeva et al., 2016, 2019). As can seen from Tables 4–6, whole-genome sequencing was performed on each of these models, except for the GC strain, in which only the expression levels of the glutamate receptor genes and the catecholamine system genes were measured.

**Table 4.**
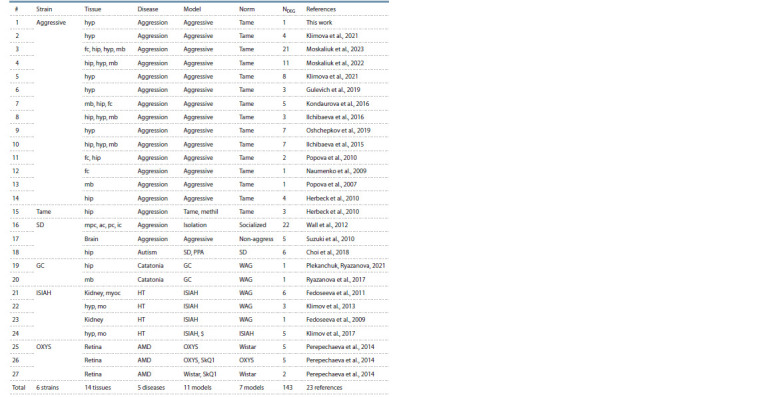
Characterization of the qPCR-inferred DEGs of the rat as a model animal in biomedicine documented in the RatDEGdb knowledge base Notе. Here and in Tables 5 and 6 : NDEG , number of DEGs. Tissues: ac, anterior cingulate; ag, adrenal gland; bmmscs, bone marrow-derived mesenchymal stromal cells; bmp, brain microvascular pericytes; bs, brain stem; fc, frontal cortex; hip, hippocampus; hyp, hypothalamus; ic, infralimbic cortex; lvcp, lateral ventricular choroid plexus; mb, midbrain; mo, medulla oblongata; mpc, medial prefrontal cortex; mt, midbrain tegmentum; myoc, myocardium; PAG, periaqueductal gray matter; pc, prelimbic cortex; po, prefrontal cortex; rc, renal cortex; rm, renal medulla. Diseases: AD, Alzheimer’s disease; AMD, age-related macular degeneration; ARBLBD, age-related blood-liquor barrier development; CRS, cellular replicative senescence; HT, hypertension; PAH, pulmonary arterial hypertension. Models: $, Agtr1a-blocker; PPA, propionic acid; SkQ1, Skulachev’s antioxidant.

**Table 5.**
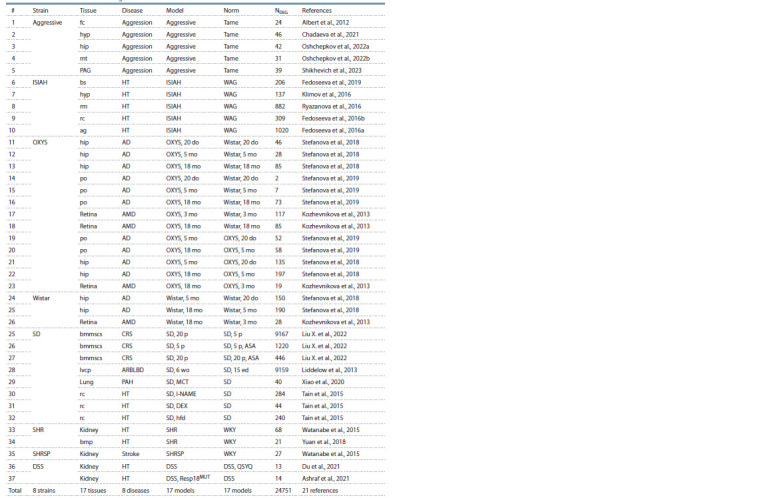
Characterization of the RNA-seq-inferred DEGs of the rat as a model animal in biomedicine documented in the RatDEGdb knowledge base Notе. Models: ASA, aspirin; do, days old; DEX, dexamethasone; ed, embryonic days; hfd, high-fructose diet; l-NAME, NG-nitro-l-arginine-methyester; MCT, monocrotaline; Resp18MUT, mutant variant; mo, months old; p, passage old; QSYQ, Chinese traditional medicine prescription Qi-Shen-Yi-Qi; wo, weeks old.

**Table 6.**
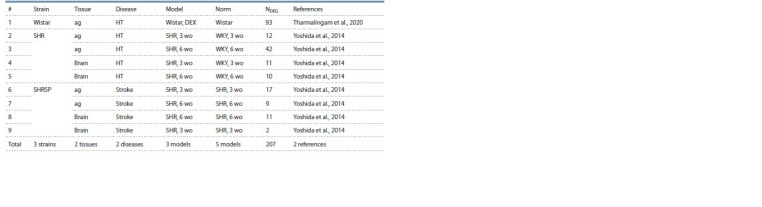
Characterization of the microarray-inferred DEGs of the rat as a model animal in biomedicine documented in the RatDEGdb knowledge base Notе. Models: wo, weeks old.

The existing biomedical databases intended for studying human diseases are normally focused on the information on the human genome (Stenson et al., 2014; Singh et al., 2018; Sun et al., 2022) and contain primary transcriptome information. RatDEGdb is novel in that it supplements biomedicinebased whole-genome experimental data on rat DEGs with clinical data on equal changes in the expression levels of their human homologs in patients, for further use of all these data in personalized medicine. With a new capability that enables the researcher to compare pathogenic changes in gene expression in humans and model animals, RatDEGdb can be useful in addressing problems in systems biology and clinical medicine.

## Conclusion

The RatDEGdb knowledge base is a collection of experimental data and a toolkit for interactive analyses in genomic research into diseases, such as Alzheimer’s disease, autism, hypertension and some others. We are planning to continue updating RatDEGdb by adding new information on gene expression in rats as model objects of human diseases and annotating the DEGs with pieces of works on equal changes in the expression levels of their human homologs in patients.

## Conflict of interest

The authors declare no conflict of interest.
